# Quantum Interference Effect in Saturated Systems: Effect of Anchoring Group Position on Conductance of Bipiperidines and their in‐situ Derived Dithiocarbamates

**DOI:** 10.1002/anie.202513806

**Published:** 2025-09-23

**Authors:** Umar Rashid, Abdalghani H. S. Daaoub, PA. Sreelakshmi, Sara Sangtarash, Hatef Sadeghi, Veerabhadrarao Kaliginedi

**Affiliations:** ^1^ Department of Inorganic and Physical Chemistry Indian Institute of Science Bangalore 560012 India; ^2^ Quantum Device Modelling Group School of Engineering University of Warwick Coventry CV4 7AL UK

**Keywords:** Bipiperidine conductance, in‐situ thiocarbamylation, *Saturated (σ) molecular structures*, Quantum interference, Quantum Transport, Single molecular conductance

## Abstract

This study investigates quantum interference in σ‐only molecular systems, focusing on the impact of anchoring group nature and its position. Using a combined approach of mechanically controlled break junction experiments and quantum transport calculations, we demonstrate that *meta*‐connected σ‐conducting bipiperidine‐based molecular junctions exhibit higher conductance than para‐connected ones. This trend is opposite to that of the trend typically observed in π‐molecular systems, indicating the existence of distinctly different quantum interference phenomenon in σ‐only molecular systems. Further, we show that anchoring group modifications achieved through in‐situ dithiocarbamylation significantly alters the conductance and decay factors. We also elucidate the mechanism behind multiple conductance features observed in aliphatic bipiperidines with cyclic secondary amine and dithiocarbamate anchoring groups. This work demonstrates σ‐quantum interference in bipiperidine‐based molecular systems and highlights how changes in junction configuration, nature of the anchoring group and its position can be used to control conductance in σ‐only molecular systems.

## Graphical abstract

This study illustrates distinct quantum interference effect in saturated systems as compared to their unsaturated counterparts and the origin of multiple conductance features observed in single molecule conductance experiments of different isomers of bipiperdine and their in‐situ generated dithiocarbamates.

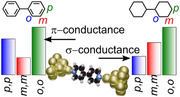

## Introduction

**Figure 1:**
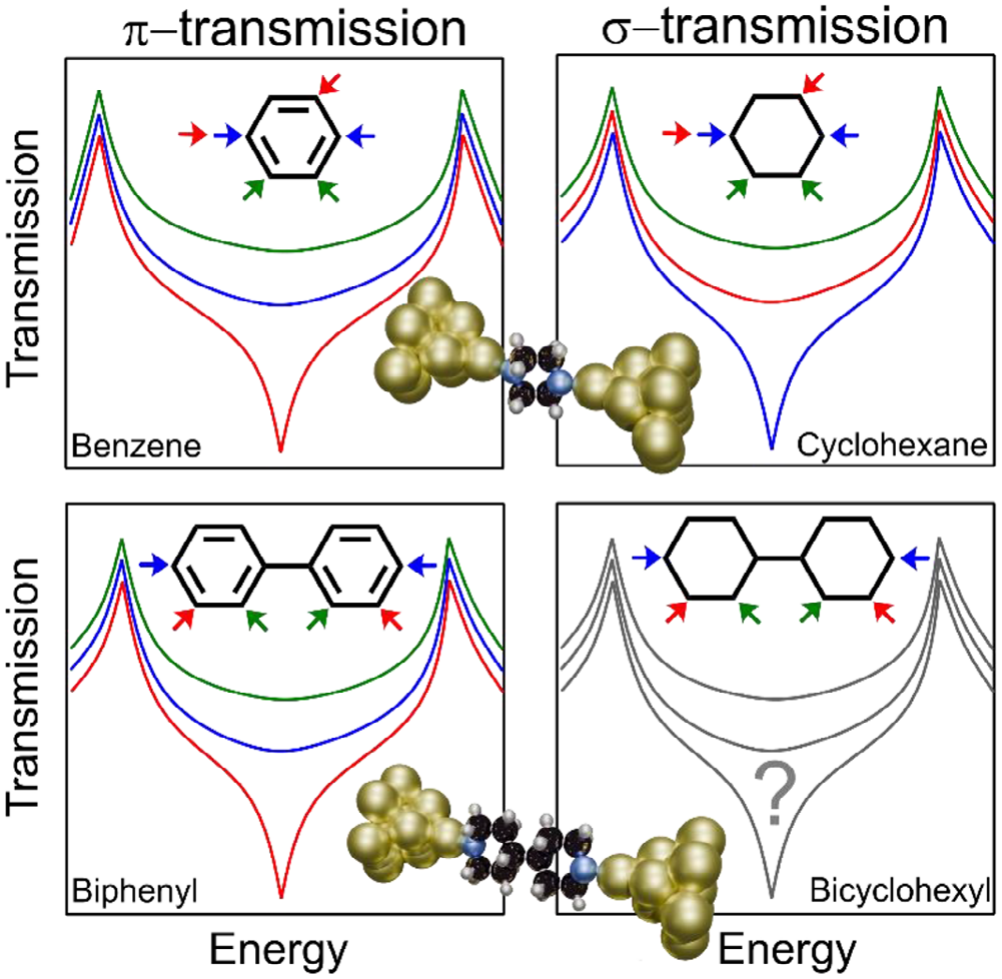
Quantum interference (QI) pattern in π and σ transmission channels in single and double ring molecular structures. The colored arrows indicate the anchoring group positions with each color corresponding to the color of the theorized transmission curve. The inserts indicate the molecular junction geometry with *para*‐anchoring groups.

Molecular electronics holds a great promise for technological advancements toward faster, energy‐efficient, and denser electronic circuitry.^[^ ^1^, ^2^, ^3^, ^4^ ^]^ Connecting single molecules to the electrodes to form molecular junctions and evaluating their charge transport properties is the primary goal of unimolecular electronics.^[^ ^4^, ^5^ ^]^ Formation of stable molecular junctions involve physisorption and/or chemisorption interactions of the anchoring group of the molecule with that of the metal apex.^[^ ^6^, ^7^ ^]^ This both broadens and shifts the molecular energy levels of the molecule with respect to its non‐binding gaseous state.^[^ ^1^, ^6^, ^8^ ^]^ While strong coupling and low‐lying frontier orbitals typically enhance conductance in coherent transport,^[^ ^9^ ^]^ however is not always guaranteed.^[^ ^1^, ^10^, ^11^, ^12^, ^13^, ^14^ ^]^ Other factors like Quantum interference (QI) effects play an important role, in deciding the net charge transport property of the molecular junctions as there are multiple conduction pathways in a molecular junction.^[^ ^10^, ^15^, ^16^ ^]^ The interference of electronic waves traveling through different conduction pathways either constructively (CQI) or destructively (DQI), significantly influences the overall electronic and thermoelectric transport properties of the molecular junction (Figure 1).^[^ ^10^, ^12^, ^17^, ^18^, ^19^, ^20^, ^21^, ^22^, ^23^, ^24^, ^25^, ^26^ ^]^ Whereas a number of rules has been set to predict the presence or absence of DQI effect in different π‐conjugated molecular systems,^[^ ^13^, ^14^, ^20^, ^27^, ^28^, ^29^ ^]^ similar rules for σ‐only systems are lacking even though Quantum interference effects are relevant in all saturated and unsaturated molecular devices.^[^ ^13^, ^30^ ^]^ This is partially due to the fact that in π‐conjugated systems which have been most explored in single molecular conductance experiments, the transport is mostly governed by the frontier molecular orbitals which in these systems are π‐orbitals. At times it is relatively easier to detect DQI as it results in antiresonances near fermi energy in certain systems which manifests as very low conductance during single molecular conductance experiments.^[^ ^10^ ^]^ However it is important to consider in all systems that may or may not show antiresonance features near fermi level.

Traditionally quantum interference (QI) has been linked to nodal structures in delocalized frontier in π‐systems.^[^ ^27^ ^]^ In these π‐systems, destructive interference is predictable from graphical or Hückel‐based symmetry rules, with antiresonances typically aligned near the Fermi energy due to orbital phase cancellation. It is well established through several theoretical and experimental demonstrations.^[^ ^31^, ^32^ ^]^ While interference effects in π‐conjugated systems are well documented,^[^ ^12^, ^13^, ^27^, ^28^ ^]^ their potential for achieving ultralow conductance molecular junctions is often limited by parasitic σ‐transport channels.^[^ ^10^, ^33^ ^]^ However the nature of σ‐transport is different from that of the π‐transport due to more delocalization in latter than former and it manifests in occurrence of DQI effect as well (Figure 1).^[^ ^10^, ^11^, ^15^ ^]^ Several studies have shown that destructive quantum interference in pure σ‐systems (σ‐DQI) like in shorter (bi)cyclic alkane and silane backbones, DQI can be realized by permethylation^[^ ^11^ ^]^ or heteroatom substitution^[^ ^34^ ^]^ with *para* substituted metal‐anchoring groups(Figure 1). For larger saturated systems, cyclizing alkane chains, which enforces gauche conformations can also induce DQI effects.^[^ ^33^ ^]^ Similarly, sila‐adamantanes which are molecular analogues of crystalline silicon unit cells, exhibit σ‐DQI which can be mechanically modulated due to the C3 symmetry at the siladiamondoid bridgeheads enforced by the dimethylsilylene bridge.^[^ ^35^ ^]^ Also, the effect of position of anchoring group on σ transmission has been demonstrated indirectly utilizing π‐conjugated system like bipyridine^[^ ^20^ ^]^ and carboxy terminated pyridines systems.^[^ ^15^ ^]^ In strict contrast to π transmission where *meta* isomers show lower conductance than their *para* counterparts, the σ transmission shows an opposite trend, here the *para* isomer shows lower conductance than meta forms(Figure 1).^[^ ^15^, ^20^ ^]^ While, this trend was obtained indirectly using π‐conjugated systems by suppressing their π‐electron transport transmission,^[^ ^15^, ^20^ ^]^ the direct probing of this effect in σ‐only systems has not yet been illustrated in larger saturated systems. Probing the effect of position and nature of anchoring group on DQI effect in purely σ‐only systems is important in two ways, first the structure of purely saturated systems is non planar, thus better chances of occurrence of DQI effect due to gauche like configurations at certain carbon centers, as the dihedral angle is primarily structural parameter to determine interference in cyclic saturated systems.^[^ ^33^, ^36^, ^37^ ^]^ Secondly, the effect of mixed σ‐π transmission on overall conductance is also eliminated. This is also important in the universal understanding of DQI effect in σ only systems, a step toward deriving analogues quantum circuit rules for these σ‐transmission.

In this paper, we experimentally and theoretically demonstrate the manifestation of QI in σ‐systems and the rules governing it, showing that the QI effect can differ in σ‐systems compared to π‐conjugated systems. This work integrates Mechanically controlled break‐junction (MCBJ) experiments using custom developed instrumentation, in‐situ chemical transformation (dithiocarbamylation) of anchoring groups, Quantum transport modelling using DFT and statistical tight‐binding (TB) frameworks. Using this combined experimental and theoretical approach, we explored the role of anchor position, orbital symmetry, and geometric flexibility on single molecular conductance of saturated molecular junctions formed by aliphatic counterparts of bipyridines called bipiperidines. By probing the different isomeric forms of this saturated system, we directly demonstrated the effect of connectivity and anchoring group position on their conductance. We demonstrated a reversal in the canonical conductance trend: para < meta < ortho for these σ‐systems, which contrasts with the well‐known trend in π‐systems (meta < para < ortho). Further, the occurrence of DQI effect depends on the nature of the anchoring groups as well,^[^ ^15^ ^]^ as differential binding can shift the frontier orbitals thus effectively changing the interference pattern. We sought to investigate this effect by probing the molecular conductance of dithiocarbamylated forms of these bipiperidines. By performing in‐situ dithiocarbamylation, we added thiocarbamate groups to the existing secondary amine anchors. This way the anchoring group is repositioned out of the cyclic ring adding the advantage of probing the effect of anchoring group position in and outside of the ring on DQI effect. We also extracted decay constants (*β*) of each conductance plateaus and master conductance curves that deconvoluted the influence of configurational changes and orbital overlap in σ‐frameworks on their molecular conductance, enabling further mechanistic insights into their conductance trends and QI behaviour.

## Results and Discussion

All the single molecular conductance experiments were performed using our custom developed break junction setup described in our previous studies.^[^ ^38^, ^39^ ^]^ Break junction techniques involve trapping a molecule in between atomically sharp electrodes while maintaining a constant potential across the interelectrode gap. This results in a small current across the tunneling gap, with the electrically connected molecular orbitals acting as channels for charge transport. As the binding geometry and molecular configuration change with each junction and considering the stochastic nature of the junction formation and breaking,^[^ ^40^ ^]^ numerous such metal molecule metal junctions are created repeatedly to map the average conductance behavior of the target molecule. Further details about the instrumentation, experimental procedure, analysis and plotting are explained in SI and our previous studies.^[^ ^38^, ^41^ ^]^

**Figure 2:**
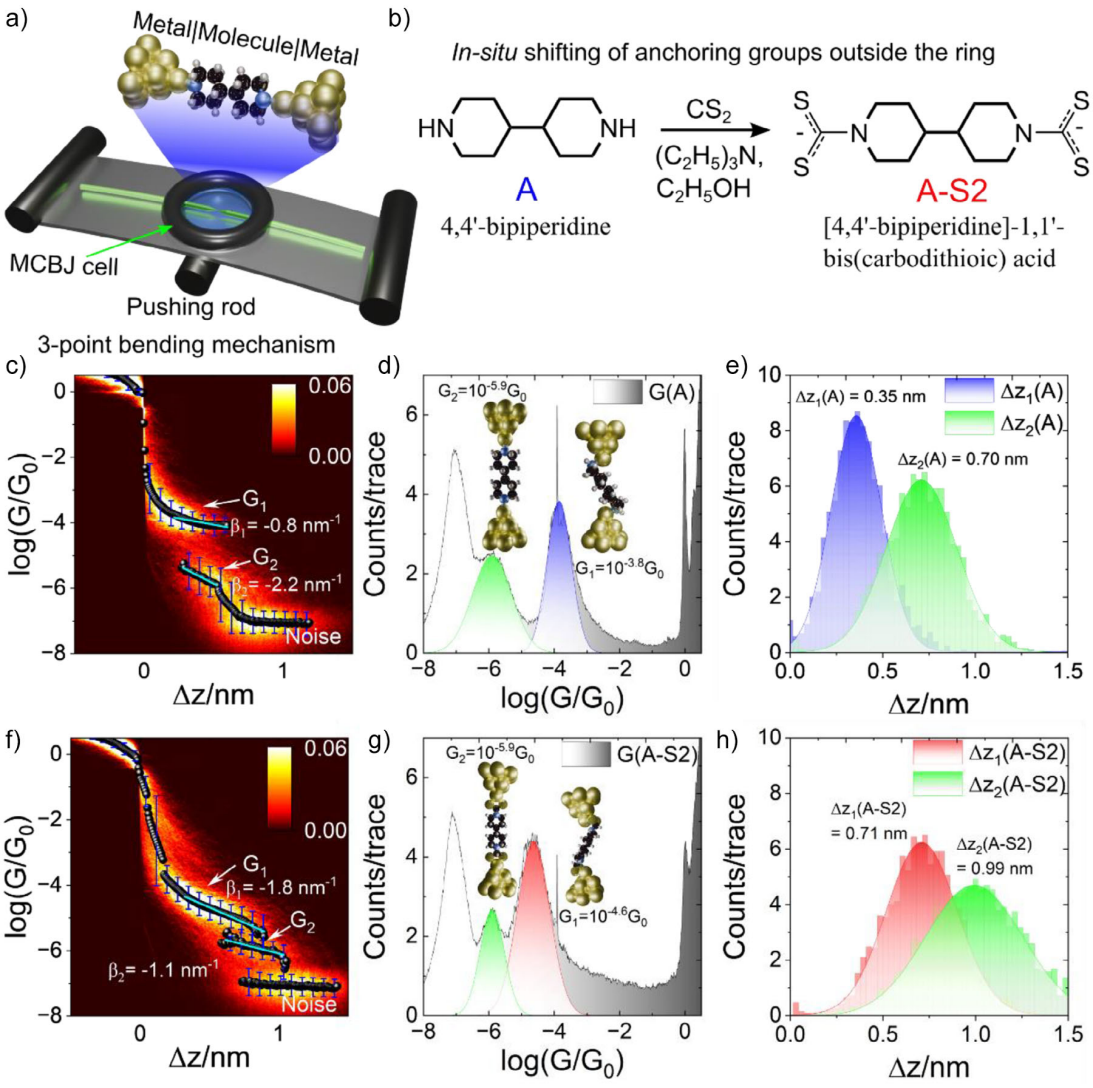
a) Schematic representation of 3‐point bending mechanism employed in Mechanically controlled break (MCBJ) technique, b) Dithiocarbamylation of 4,4`‐bipiperidine‐Molecule A, c) 2D conductance‐distance histogram of A at 100 mV applied bias. Corresponding d) 1D conductance (Insets depict the junction configurations for end‐to‐end and sideways binding) and e) plateau length histograms, f) 2D conductance‐distance histogram of A‐S2 at 100 mV applied bias. Corresponding g) 1D conductance (Insets depict the junction configurations for end‐to‐end and sideways binding) and h) plateau length histograms. The black balls and corresponding error bars in c) and f) represent the master curve extracted from respective 2D histogram, green lines represent the linear fit giving the decay factor(*β*) represented on the plots.

First, we measured the single molecular conductance of the 4,4`‐bipiperidine (A, Figure 2b) by taking its 0.2 mM solution (in THF:TCB: ¼ v/v solvent) in the MCBJ cell. More than 1500 conductance‐distance traces were recorded, and the results are plotted in Figure 2c–e. Figure 2c shows the 2D conductance‐distance histogram of molecule A recorded at an applied bias of 100 mV across the junction. The 2D conductance‐distance histogram depicts the variation of conductance of the molecular junction as a function of the interelectrode gap. At very high conductance values, we see a sharp peak corresponding to the conductance of a single Au atom (quantum unit of conductance = 1 *G*_0 _= 77.5 µS). This is followed by a sharp drop in conductance due to the Au snapback region. This originates from the snapping of Au electrodes from each other to about 0.5 ± 0.1 nm, from the initial metal‐metal contact.^[^ ^42^, ^43^ ^]^ Following the snap‐back region, we see a conductance plateau corresponding to the first single molecular conductance feature of A molecule (*G*_1_ = 10^−3.8^ *G*_0_). The conductance plateau shows the change in conductance of the metal|molecule|metal junction as the molecular junction is stretched.

The corresponding 1D conductance histogram is shown in Figure 2d. It is evident from the 2D and 1D conductance histograms (Figure 2c,d) that there is another conductance feature at lower conductance than *G*_1_ (*G*_2_ = 10^−5.9^ *G*_0_). In the case of aromatic molecular systems, usually, the lower conductance peak other than the conductance of monomer junctions corresponds to that of the junctions formed by intermolecular association and involves more than a single molecule as a connection between electrodes.^[^ ^44^ ^]^ These dimer junctions presents with an interelectrode gap distance more than that of the single molecular length. From the plateau length histogram (Figure 2e), we can interpret that *G*_1_ corresponds to a molecular junction with an interelectrode gap of about 0.85 (±0.1) nm and *G*_2_ to about 1.20 (±0.1) nm (after adding Au snapback to plateau lengths obtained experimentally). Considering the end‐to‐end molecular length of A (Figure S13, computed electrode‐electrode separation *z*_1 _= 0.72 nm_,_ *z*_2_ = 1.23 nm), *G*_2_ peak arises to a fully stretched junction while as *G*_1_ due to the junction configurations with lower gap distance than the molecular length (see insets in Figure 2d). We presume that *G*_1_ conductance plateau is due to the binding of the molecule at some angle to the electrodes (further confirmed by transmission calculations, see Figure S13). For molecule A, the anchoring groups are a part of the cyclohexane moieties. To evaluate the conductance response when the anchoring group is outside the aliphatic ring, we performed in‐situ dithiocarbamylation of molecule A in the MCBJ cell. This was done following the procedure as described in the literature.^[^ ^45^, ^46^, ^47^ ^]^ Briefly a small amount of CS_2_ and (C_2_H_5_)_3 _N solutions(100 mM prepared in THF) were added to the MCBJ cell along with 5 µL of C_2_H_5_OH containing A in the same sequence to get the resultant concentration of A:CS_2_:(C_2_H_5_)_3 _N to 1:2:2. This leads to a spontaneous irreversible reaction of CS_2_ with the secondary amine yielding a dithiocarbamate derivative of A (A‐S2, Figure 2b). The conductance experiments were started immediately after the addition of the reaction mixture. Single molecule conductance results for the dithiocarbamate product(A‐S2) are plotted in Figure 2f–h. The change in both the most probable conductance values and the plateau features is apparent after comparing Figure 2c,f. This is due to the change of the anchoring groups (secondary amines to dithiocarbamates) and the increase in molecular length with the addition of two CS_2_ groups to molecule A. The change in conductance of molecule A after dithiocarbamylation is further evident from the 1D conductance histograms (Figure 2d,g) and the increase in molecule length from plateau length histograms (Figure 2e,h). Further, the conductance experiments show that the reaction is spontaneous, and the product formation is achieved immediately after the addition of the reaction mixture, as described in the literature.^[^ ^45^, ^46^ ^]^ The A‐S2 product also shows two different conductance plateaus in the 2D conductance‐distance histogram(Figure 2f). The origin of these two plateaus is similar to that of its parent bipiperidine reactant (see insets Figure 2g), i.e., difference in binding geometry considering the plateau lengths of the two conductance plateaus and computed molecular length of A‐S2 (See Figure S14). However, we can see that *G*_1_ conductance decreases upon dithiocarbamylation (from 10^−3.8^ *G*_0_ for A to 10^−4.6^ *G*_0_ for A‐S2) but G_2_ (10^−5.9^ *G*_0_) almost remains the same. This is also confirmed by the quantum transport calculations below. Further, the experimental plateau lengths match well with the theoretically calculated ones for both the reactant and the product.

From the above‐mentioned experimental observation, it is evident that different conductance features can arise in aliphatic systems due to the different anchoring geometry in these systems as the junctions are stretched. Although a similar behavior can be seen in aromatic systems like in bipyridines,^[^ ^48^, ^49^ ^]^ but there the multiple conductance features arise due to the involvement of the sidewise electrode‐π interactions. This leads to a small conductance difference between the different conductance plateaus, but in σ systems (as seen above), the large difference in conductance between these features is due to larger dependence of single molecular conductance on interelectrode gap than in aromatic systems. This also illustrates that even though the π orbitals are not available, different conductance features can arise due to the junction configuration change (placement of molecule with respect to electrodes) in addition to other geometrical and conformational changes. To further evaluate the conductance behavior of different features for both anchoring groups, we calculated the master curves from the overall 2D histograms (Figures 2c for A, 2f for A‐S2). This was achieved by fitting the Gaussian function at each of the x value of the 2D histogram, and the peak value gives the most probable conductance value at that electrode gap distance (Δ*z*). Following the same procedure for all Δ*z* values, a master curve is plotted as seen in Figure 2c,f as black balls. Fitting the master curve conductance plateau with a linear function gives the decay constant (*β*) for that feature which relates to the change in conductance with increase in interelectrode distance as (*d*(log(G/G_0_))/*d*z = Ae*^βz^*) where, *G* is the conductance and *z* is interelectrode gap distance). A large negative value of *β* indicates that the junction configuration is more susceptible to conductance change as the binding angle changes, and the vice‐versa is also true. From these plots, we can see that the secondary amine (NHR_2_) group shows smaller change in conductance as compared to the CS_2_ group when the binding is sideways (at smaller angles, *β_1_* = −0.8 versus −1.8 nm^−1^), and the trend reverses for an end‐to‐end binding (for larger angles, *β_2_* = −2.2 versus −1.1 nm^−1^). This indicates that smaller anchoring angles (side‐on binding) enhance orbital overlap between anchoring group lone pair (N atoms of bipiperdine) and gold electrode orbitals, resulting in stronger coupling and a slower decay of conductance with stretching (smaller |*β*|). Conversely, larger anchoring angles (end‐to‐end binding) reduce this overlap, making the junction more sensitive to stretching, resulting in larger |*β*| value. After dithiocarbamylation, as the more extended geometry results in stronger binding than side‐on binding, the |*β*| trend reverses. Thus, from the decay parameter (|β|) trends it can be inferred that in‐situ dithiocarbomylation is altering the spatial electron density distribution of the anchoring group (from secondary amine to dithiocarbomate) leading to distinctly different optimum geometry for the effective charge transport.

**Figure 3:**
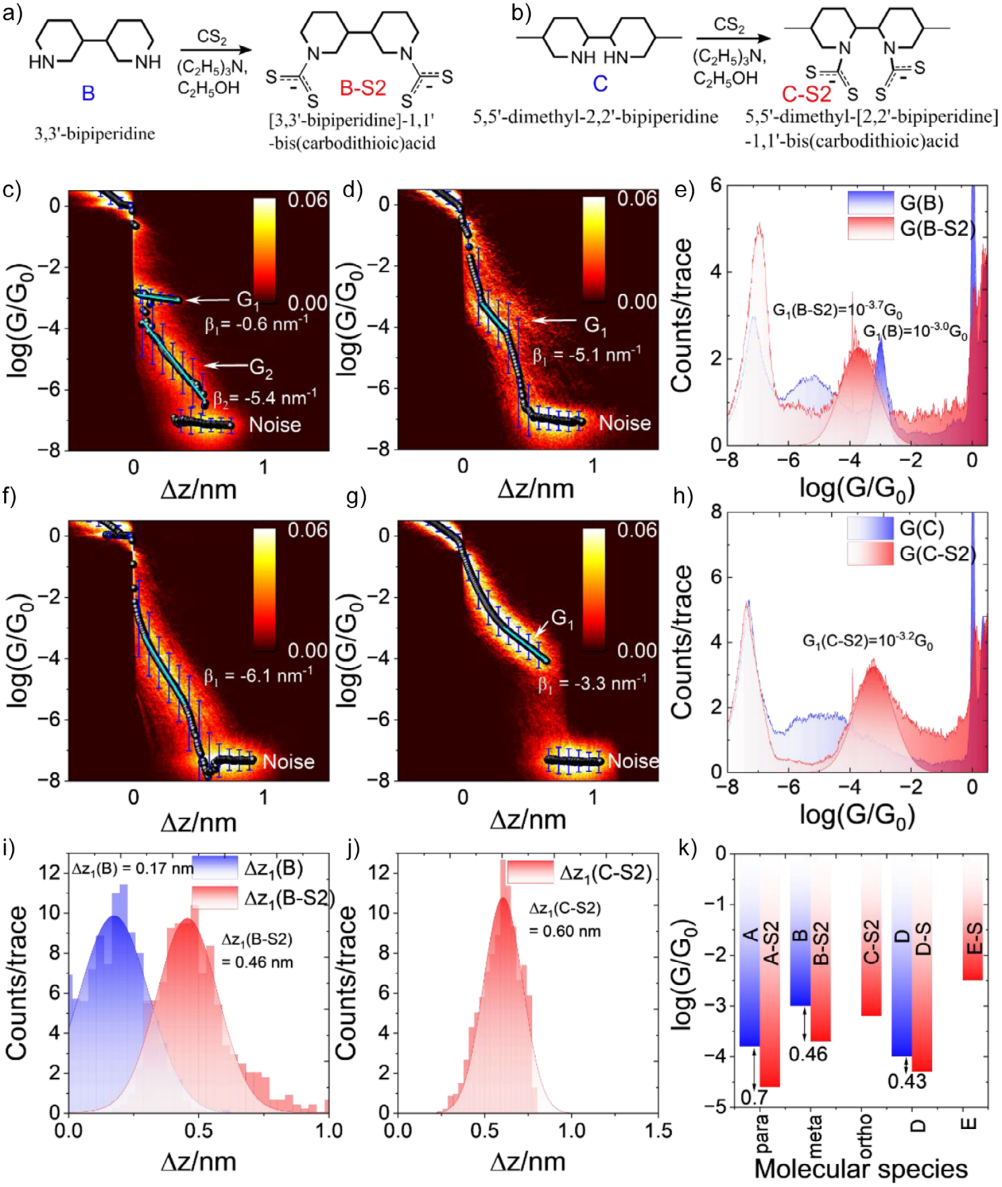
Dithiocarbamylation of a) *meta*‐bipiperdine‐B, b) *ortho‐*bipiperdine‐C. 2D conductance‐distance histogram of c) *meta*‐bipiperdine‐B at 100 mV applied bias and its d) dithiocarbamate derivative‐B‐S2, e) Corresponding 1D conductance histograms of B and B‐S2. f) 2D conductance‐distance histogram of *ortho*‐bipiperdine‐C at 100 mV applied bias and its g) dithiocarbamate derivative‐C‐S2, h) Corresponding 1D conductance histograms of C and C‐S2. (i) Plateau length histograms corresponding to *meta*‐bipiperdine and its dithiocarbamate derivative. j) and *ortho*‐bipiperidine‐C‐S2. k) Conductance trend for all the molecular systems probed in this study. Notice the decreasing conductance from *ortho* to *meta* to *para* derivatives for bipiperidines for both amine and dithiocarbamate anchoring groups.

Next, to show the effect of the position of the anchoring group in these aliphatic systems on the conductance behavior, we measured the conductance of the meta (Figure 3a) and ortho (Figure 3b) analogs of A and their dithiocarbamate derivatives. The results are shown in Figure 3c–j. The *meta*‐analogue (B) shows higher conductance (10^−3.0^ *G*_0_) as compared to *para*(A) (10^−3.8^ *G*_0_) and a smaller change in conductance with the interelectrode gap (*β_1_* = −0.6 nm^−1^ for molecule B). This smaller change is due to the different geometrical freedom available to the systems when it is trapped between the electrodes in *meta* and *para* derivatives. For *meta*, the *G*_2_ corresponds to the tunneling through the solvent as assumed from its decay factor (*β_2_* = −5.4 nm^−1^) which is close of that of tunneling through the solvent.^[^ ^38^ ^]^ This is reasonable considering the higher steric hindrance offered to the electrodes at higher binding angles. The dithiocarbamate product(B‐S2) also shows a single conductance peak (Figure 3d) with conductance 10^−3.7^ *G*_0_ and high *β* (*β_1_* = −5.1 nm^−1^). For the ortho (C, Figure 3f) we didn't see any binding of the molecules to electrodes but instead blank tunneling traces further confirmed by the decay factor (*β_1_* = −6.1 nm^−1^). This might be due to the steric hindrance offered to binding to the electrodes at the *ortho* position of anchoring groups. However, if we protrude out the anchoring groups the hindrance should be relieved enough for them to bind to electrodes, which is the case we observed for its dithiocarbamate derivative (Figure 3g) which gives a conductance peak at 10^−3.2^ *G*_0_. This is an additional benefit of performing in‐situ dithiocarbamylation reaction, if a molecule is too small to be measured directly using break junction experiments it can be elongated by the in‐situ reaction so that conductance of the resultant product can be measured. However, the conductance behavior of C‐S2 is radically different for the entire trace. First, we don't see a sharp Au snapback region, and when electrodes are stretched apart, the system geometry radically changes, thus leading to a larger change in conductance (*β* = −3.3 nm^−1^ for C‐S2). From the above experimental observations, we see the conductance trend as follows A < B (*para < meta*) for secondary amines and A‐S2 < B‐S2 < C‐S2 (*para < meta < ortho*) for dithiocarbamates for high conductance peak (*G*_1_), which indicates a different quantum interference trend is observed in these systems as compared to the aromatic systems, where conductance follows the trend(*meta < para < ortho*).^[^ ^50^ ^]^ A similar trend for the lower conductance peak(*G*_2_) cannot be evaluated as only para isomer (A) shows two molecular conductance peaks (*G*_1_ and *G*_2_).

We also performed similar experiments with molecule D (Figure S8A). This has an aliphatic system only on one side and undergoes dithiocarbamylation on that side only (Figure S8A). The pyridine group is not susceptible to this reaction.^[^ ^46^, ^47^ ^]^ For this system, we see a small change in conductance(10^−4^ to 10^−4.3^ *G*_0_) from the reactant (D) to the product (D‐S), which is reasonable as only one CS_2_ group is added to the reactant (Figures S8–S10). For both the reactant (D) and the product (D‐S), we see two conductance features similar to A (Figures S9 and S10). These experiments also demonstrate that the break junction technique can be used to probe reactions involving the change of the anchoring groups in addition to transformation of the main functional unit as illustrated in literature.^[^ ^51^, ^52^ ^]^ It is also evident from above conductance measurements that no reactant conductance feature is seen in product conductance experiments, this illustrates that the binding of secondary amines to metal electrodes doesn't make them resistant to dithiocarbamylation. Had there been any unreacted reactant species left after dithiocarbamylation, the corresponding conductance features would have been observed. We also measured a molecule with primary amine on one side and secondary amine on another (E, Figure S8B). Here, the dithiocarbamylation is expected on the secondary amine side only. From the Figures S11 and S12 it is evident that only product gives conductance plateau(E‐S) but not reactant (E) this might be due to the short molecular length that falls in the Au snap‐back region. As the molecular length is increased by dithiocarbamylation and the anchoring group changes to stronger dithiocarbamate we see a peak behavior similar to C. The large conductance decay factor (*β* = −3.5 nm^−1^) for E‐S is indicative of large geometrical transformation as the molecular junction stretches. Figure 3k shows the conductance behavior of all the molecular species probed in this study. And it can clearly be seen that the conductance for bipiperidines follows the trend (*para < meta < ortho*) similar to what has been observed for σ‐transmission for smaller cyclic and bicyclic systems.^[^ ^10^, ^15^, ^34^ ^]^

**Figure 4:**
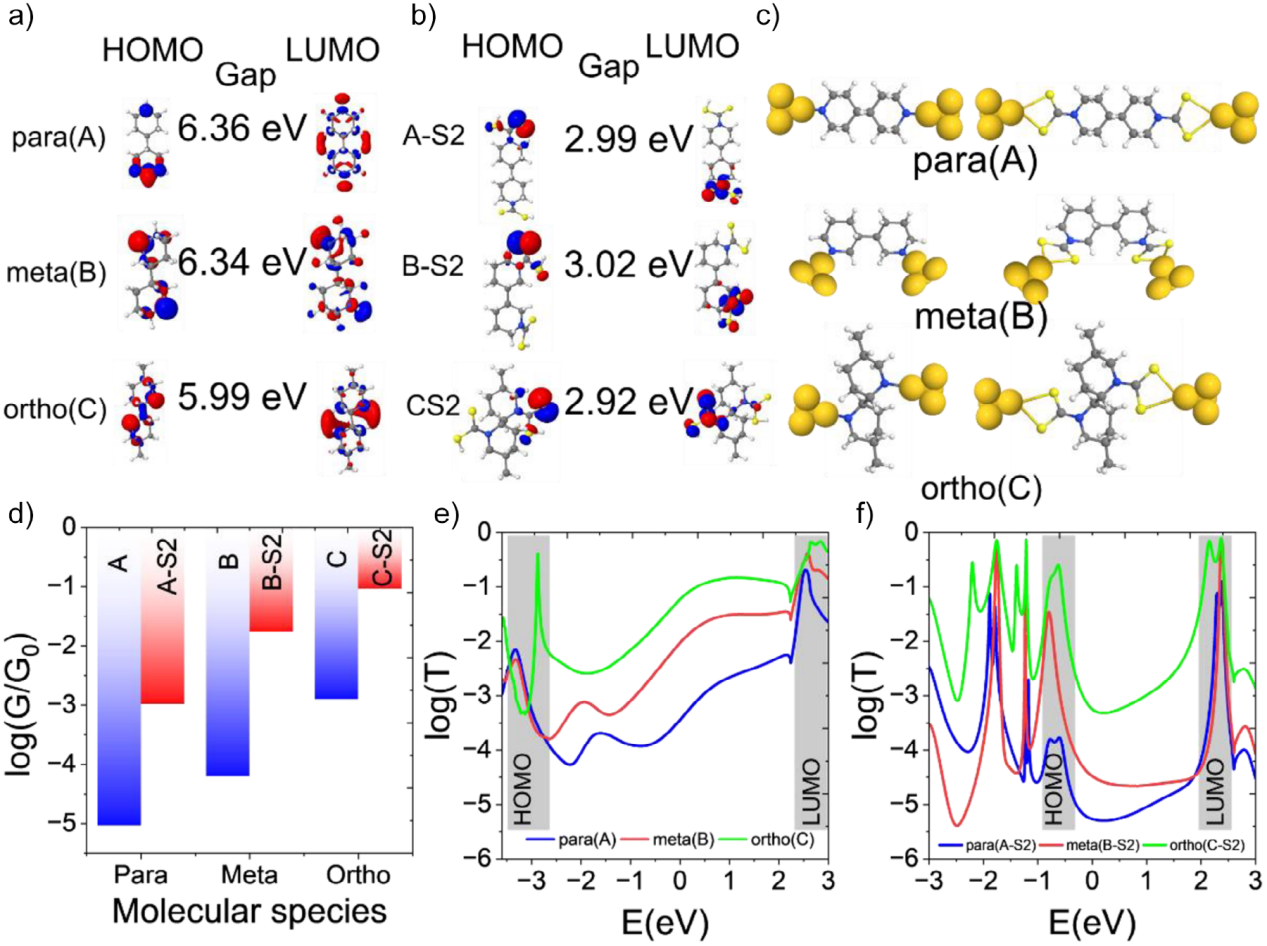
DFT calculations of molecular structures of bipiperidines and their dithiocarbamate derivatives. a), b) Molecular orbitals (HOMO and LUMO) for the *para, meta*, and *ortho* configurations of bipiperidine and their dithiocarbamylated products. c) Optimized junction geometries of A, B, C and A‐S2, B‐S2, C‐S2 between gold electrodes. d) Bar graph representing the average room‐temperature conductance over a Fermi energy range (*E*_F_ = −0.5 to 1.5 eV), corresponding to e), f) shows transmission coefficients for the *para*, *meta*, and *ortho* configurations of bipiperidine and their dithiocarbamylated products, respectively, highlighting the impact of structural and parametric variations on electron transport properties.

To better understand the electronic and transport properties of the studied molecular junctions, we performed first‐principles calculations using SIESTA^[^ ^53^ ^]^ implementation of density functional theory (DFT) (see Supporting Information for details). Initially, we optimized the isolated molecules in the gas phase to identify their ground‐state conformations. These optimizations show that the chair conformation of the cyclohexane ring is energetically more favourable than the boat conformation (Figure S20); accordingly, all junction models were constructed from the chair geometry. We then calculated the molecular orbitals and energy levels. The HOMO and LUMO distributions for various structures (Figure 4a) reveal that the wavefunction is predominantly localized on the nitrogen atoms in the *para*, *meta*, and *ortho* configurations of bipiperidine. This behaviour contrasts with typical π‐systems, where the wavefunction is more delocalized across the entire molecule (see Table S3). The HOMO‐LUMO gap for all configurations is relatively large, ∼6.0 eV (Figure 4a). For their dithiocarbamate derivatives (Figure 4b), the wavefunction is primarily localized on the sulphur atoms rather than being evenly distributed across the molecule. Notably, dithiocarbamates exhibits a significantly lower HOMO‐LUMO gap of ∼3.0 eV, (for more information see Table S2).

To understand the charge transport properties of junctions, we then calculated the electrical conductance of molecules between electrodes. For this, the molecular structures were placed between gold (Au) electrodes and geometry optimization were performed using DFT. The optimized junction geometries are shown in Figure 4c. Charge transport calculations were performed for all junctions using the quantum transport code GOLLUM.^[^ ^29^, ^54^, ^55^ ^]^ This allowed for the calculation of the transmission probability of electrons with a given energy traversing through the molecule from one electrode to the other, as shown in Figure 4e,f for bipiperidines and their dithiocarbamate derivatives, respectively. The varying angles between the molecules and electrodes influence charge transfer efficiency. A smaller angle between the nitrogen atoms and gold electrodes increases the overlap between the nitrogen lone pairs and the electrode orbitals, enhancing electronic coupling and conductance. This effect leads to two distinct conductance groups, as illustrated in Figures S13 and S14. Figure 4e,f display the transmission coefficients as a function of energy for molecular structures bipiperidines and their dithiocarbamate derivatives, respectively. In bipiperidines, the *para* configuration exhibits the lowest transmission probability for a wide energy range around the DFT Fermi energy (E = 0 eV), while the *ortho* configuration shows the highest transmission probability, contrary to the trend observed in π‐systems.^[^ ^56^, ^57^ ^]^ The conductance trends are quantitatively summarized in Figure 4d, which reveals that the ortho configuration achieves the highest electrical conductance, while the *para* configuration has the lowest reinforcing the *ortho > meta > para* conductance hierarchy. Additionally, secondary amines consistently demonstrate higher conductance than their dithiocarbamate derivatives, primarily due to its shorter molecular length, thereby facilitating more efficient charge transport. These trends are in good agreement with experimental conductance measurements, demonstrating that conductance can be systematically modulated via structural and parametric engineering of molecular junctions. Furthermore, both our DFT calculations and experiment show that, unlike in π‐systems, the conductance through meta connectivity is higher than that of para. We attribute this unexpected conductance trend to QI in σ‐systems that is different from the QI in π‐systems.

**Figure 5:**
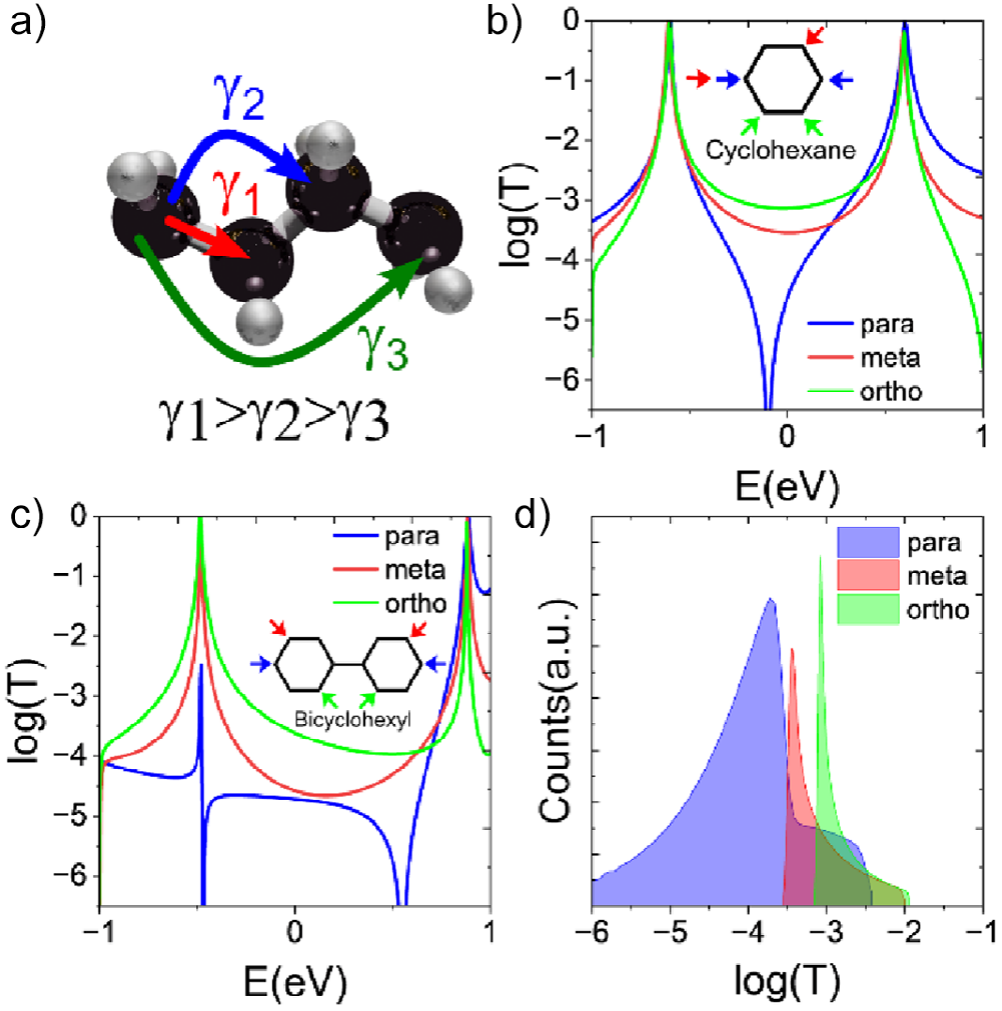
Tight‐binding (TB) model for quantum interference in σ‐system. a) TB model for interaction between σ orbitals. *γ*_1_ = 0.8γ, *γ*_2_ = 0.6*γ*, *γ*_3_ = 0.4*γ*, where *γ* = −2 eV is the coupling strength between orbitals in the leads (see methods in the Supporting Information for more details.) Connectivity dependent TB transmission function for b) cyclohexane, and c) bicyclohexyl. d) histogram of TB transmission functions for a bicyclohexyl with 15% tolerance in TB parameters.

To elucidate the QI patterns within the σ‐system, we initially analyzed the molecular orbital rules as shown in Figure 4a,b.^[^ ^29^ ^]^ This analysis revealed that these rules alone were insufficient to predict the QI effect and explain the observed conductance trends. In what follows, we use a simple tight‐binding (TB) model to demonstrate the manifestation of QI in σ‐systems. Other similar methods have been used in the literature to study σ‐systems.^[^ ^58^, ^59^, ^60^ ^]^ Here we develop the simplest TB model, depicted in Figure 5a, with one orbital per atom. In this model, each σ‐orbital interacts with its nearest, second‐nearest, and third‐nearest neighbors (see Supporting Information for details). Using this TB model, we calculated the transmission coefficients for two σ‐systems: cyclohexane (Figure 5b) and bicyclohexyl (Figure 5c). These calculations were performed for *para*, *meta*, and *ortho* connectivities, with the systems coupled to TB electrodes. With the parameters used (see Supporting Information), which were parameterized based on a Hückel Hamiltonian derived from DFT, we observed constructive QI for ortho and meta connectivities, and destructive QI for para connectivity (Figure 5b,c). Figure 5b,c show examples of transmission coefficients that display clear anti‐resonance for para connections. This anti‐resonance is highly sensitive to small changes in parameters and can be suppressed by introducing changes of even less than 15% in hopping integral values. These variations can arise in σ‐molecular junctions due to their inherent structural flexibility. Figure S19 shows the transmissions for these models by introducing 15% random perturbations to the parameters. Clearly, the transmissions specially the antiresonances for para connection are affected by this variation. However, when a histogram^[^ ^61^ ^]^ of the resulting transmission values are created, as shown in Figure 5d, the peak of this histogram, representing the most probable transmission value, shows the trend *ortho > meta > para* in good agreement with the experimental values. This demonstrates that while a clear anti‐resonance in transmission might not always be expected for a para configuration (as seen in the DFT transmissions in Figure 4e,f), the QI effect in σ‐systems for the para connection statistically leads to lower conductance compared to the meta connection. We note that the length of the shortest path for an electron to travel through the molecule from one electrode to the other increases as the connection points change from ortho to meta to para. While this path length could also contribute to the decrease in conductance, a distinct change in the interference pattern from CQI in the meta connectivity to DQI in the para connectivity in our TB model suggest that the QI plays a dominant role.

## Conclusion

This study illustrates the distinct quantum interference (QI) patterns governing charge transport in saturated molecular systems as compared to unsaturated systems. Through single‐molecule conductance measurements on cyclic secondary amines and their dithiocarbamate derivatives together with theoretical modeling, we demonstrate that aliphatic bipiperidines exhibit single molecular conductance trend: *para* < *meta* < *ortho*, contrasting sharply with aromatic systems where *meta* configurations typically show suppressed conductance due to destructive π‐interference. The absence of a *meta* effect in these larger σ‐only systems illustrates a fundamental distinction of transport rules between saturated and unsaturated molecular systems.

This study also establishes that the observed multiple conductance features in the sigma systems arise due to configurational changes in the junction geometry during stretching, which modulate the overlap between localized σ‐orbitals and electrode. We further establish the effect of anchoring group chemistry on the conductance features of single molecular junctions: in‐situ dithiocarbamylation changes conductance decay factors (*β*) compared to secondary amines, highlighting how binding groups dictate the coupling strength. Further, the in‐situ chemical modification confirms complete conversion of bipiperidines to corresponding thiocarbamate products, similar to what has been observed using conventional analytical techniques, validating the robustness of break‐junction techniques for probing interfacial reactions.

Theoretical calculations, including DFT and tight‐binding models, validate experimental trends, attributing conductance variations to destructive QI in *para* configuration and constructive interference in *ortho/meta* configurations. Our findings provide a strategy for designing σ‐only molecular junctions with tailored electronic properties by leveraging anchoring group selection and stereochemical control. Future work could extend these principles to hybrid σ‐π systems and exploit configurational flexibility for thermoelectric applications. By bridging the gap in understanding QI effect in larger saturated systems, this work advances the rational engineering of reproducible, high‐performance molecular junctions based on σ‐only molecular architectures.

## Supporting Information

Supporting information including all the single molecular conductance data and theoretical methods and results is available for this article. The authors have cited following references within the Supporting Information.^[^ ^29^, ^38^, ^39^, ^41^, ^53^, ^54^, ^61^, ^62^ ^]^

## Conflict of Interests

The authors declare no conflict of interest.

## Supporting information



Supplementary Information

## Data Availability

The data that support the findings of this study are available from the corresponding author upon reasonable request.
