# Supplementary material for: Quantum Interference Effect in Saturated Systems: Effect of Anchoring Group Position on Conductance of Bipiperidines and their in‐situ Derived Dithiocarbamates
Source: Angew Chem Int Ed Engl. 2025 Sep 23;64(47):e202513806. doi: 10.1002/anie.202513806 (PMC12624308; doi:10.1002/anie.202513806)
Supplement: Supplementary file 1 — Supplementary Information [file ANIE-64-e202513806-s001.docx]

**Supporting Information**

Quantum interference effect in Saturated systems: Effect of anchoring group position on conductance of bipiperidines and their *in-situ* derived dithiocarbamates

Umar Rashid,^1, †^ Abdalghani H. S. Daaoub,^2, †^ PA Sreelakshmi,^1^ Sara Sangtarash,^2^ Hatef Sadeghi,^2^* and Veerabhadrarao Kaliginedi*^1^

^1^ *Department of Inorganic and Physical Chemistry, Indian Institute of Science, Bangalore 560012, India*.

*^2^ Device Modelling Group, School of Engineering, University of Warwick, Coventry CV4 7AL, U.K.*

*Corresponding Authors’ E-mail: hatef.sadeghi@warwick.ac.uk (H.S.); vkaliginedi@iisc.ac.in*

^†^ These authors contributed equally to this work.

**Contents:**

1. Materials
2. Single molecular conductance measurements and data.
3. Computational studies
   1. Computational methods
   2. Supplementary computational figures, tables and discussion
4. References
5. **Materials:**

All the molecules used in this study (4,4'-bipiperidine-**A**, 3,3'-bipiperidine-**B**,5,5'-dimethyl-2,2'-bipiperidine-**C**,4-(piperidin-4-yl)pyridine-**D**,piperidin-2-yl-methylamine-**E**) were ordered from TCI chemicals and used as obtained. We also explored 1,3-di(piperidin-4-yl)propane, 1,4'-bipiperidine,N^1^-methylpropane-1,3-diamine,N^1^,N^3^-dimethylpropane-1,3-diamine also ordered from TCI chemicals but these didn’t show any clear conductance features in conductance experiments due to their shorter molecular length. The solvents that were used to prepare molecular solutions for conductance experiments (inhibitor free Tetrahydrofuran (THF,99.99%) and 1,2,4-trichlorobenzene (TCB,99 %) were ordered from Sigma Aldrich. Carbon disulfide (CS_2_) and triethylamine (C_2_H_5_)_3_N,99.5%) used to perform thiocarbamylation reactions were also acquired from Sigma Aldrich. The 0.1 mm Au wire (99.99%) for preparing break junction samples was acquired from Goodfellow. All the conductance experiments were performed in dark under ambient conditions.

1. **Single molecular conductance measurements and data:**

MCBJ technique allows reproducible formation of molecular junctions by repeatedly trapping single molecules from the molecular solution between the atomically sharp electrodes, created in-situ from a notched electrode wire. The repeated formation of molecular junctions is achieved by creating a nanogap in between the electrodes. This is realized by controlling the bending of a flexible stainless strip on which a 0.1 mm Au wire is mounted at two closely separated spots at its center by a chemically resistive epoxy resin. The strip is mounted on a three-point bending mechanism (Figure 2A) and pushed upwards in the middle; this creates a strain on the mounted electrode wire. The wire constricts at the notched place and ultimately breaks, creating an interelectrode nano-gap. By controlled bending of the SS strip, the nanogap can be modulated at Angstrom-level resolution. The bending of the strip is performed using a piezostack and stepper motor combination, details of which are given in our previous studies.^[1–3]^ The molecules present in the solution in the MCBJ cell covering the metal wire bind to the electrodes and form the molecular junction. As the electrode gap increases, the molecular junctions get stretched and ultimately break, leading to tunneling current through the solvent. Then, the SS strip is relaxed back, leading to a decrease in the interelectrode gap and ultimately to metal-metal contact. Upon detecting a metallic current, the bending is repeated again, and this way, junctions are created repeatedly. A constant bias is maintained across the electrodes, and the resulting current is measured continuously as a function of interelectrode gap distance. By creating thousands of such junctions, the conductance corresponding to breaking junctions is analyzed further and binned suitability to give various conductance-length plots. More details about the analysis and plotting is given in our previous studies.^[1–4]^

When a molecule bridges between the two electrodes, a molecular junction forms. In the absence of such an electronic connection, electrical conductance between the electrodes decays exponentially with increase in the inter-electrode gap, which is characteristic of tunneling through a solvent potential barrier. The formation of a molecular junction is indicated by a plateau in the conductance trace as the electrodes are pulled apart. The conductance of a molecule within the molecular junction can vary due to the factors like geometric variations and, fluctuations in energy levels and electronic coupling. Conductance histograms are constructed from multiple breaking traces to account for this variability. The prominent peak(s) in these histograms typically fit with a Gaussian function to evaluate the most probable molecular conductance statistically.

Below we show the conductance data for all the molecular (A-E) systems measured with 0.2 mM concentration of the molecule unless otherwise stated:


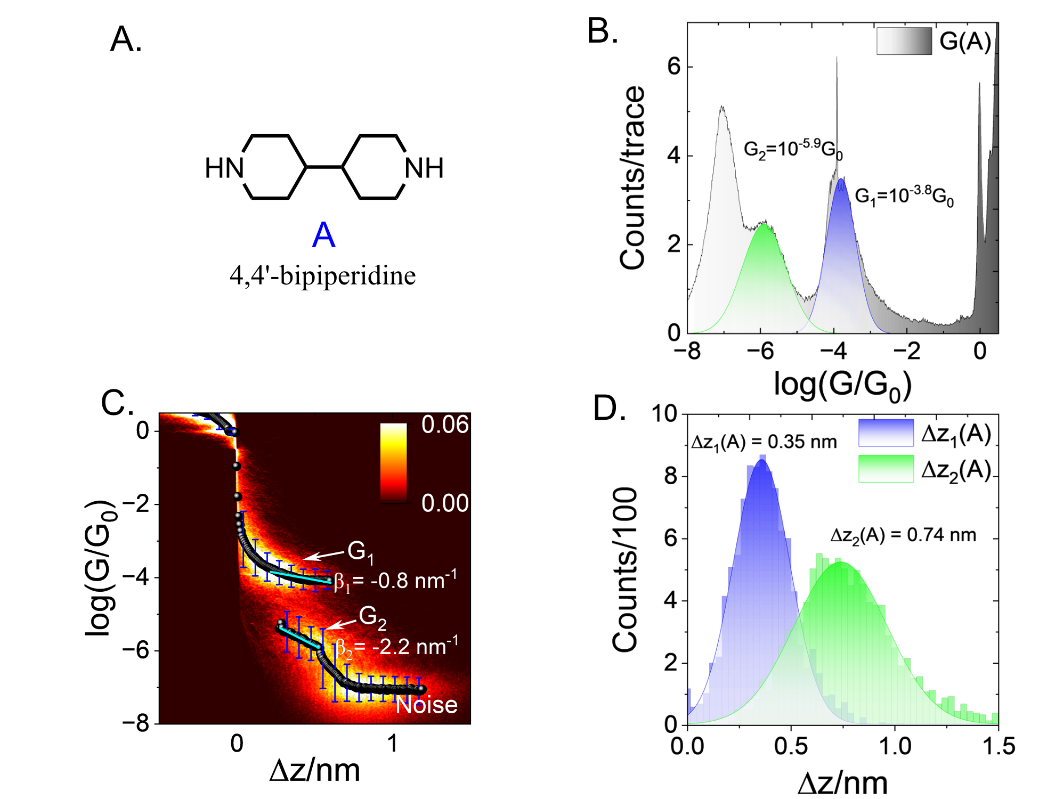


**Figure S1. A.** Molecular structure of 4,4'-bipiperidine-A molecule. **B.** 1D conductance **C.**2D conductance-distance and **D.** plateau length histograms**.** (In 2D conductance histogram, the black balls represent the Gaussian peak maximum at each of the x values, and black error bars represent the standard deviation derived from the Gaussian fitting. Purple line represents the linear fit giving the decay factor(β) represented in the plots.


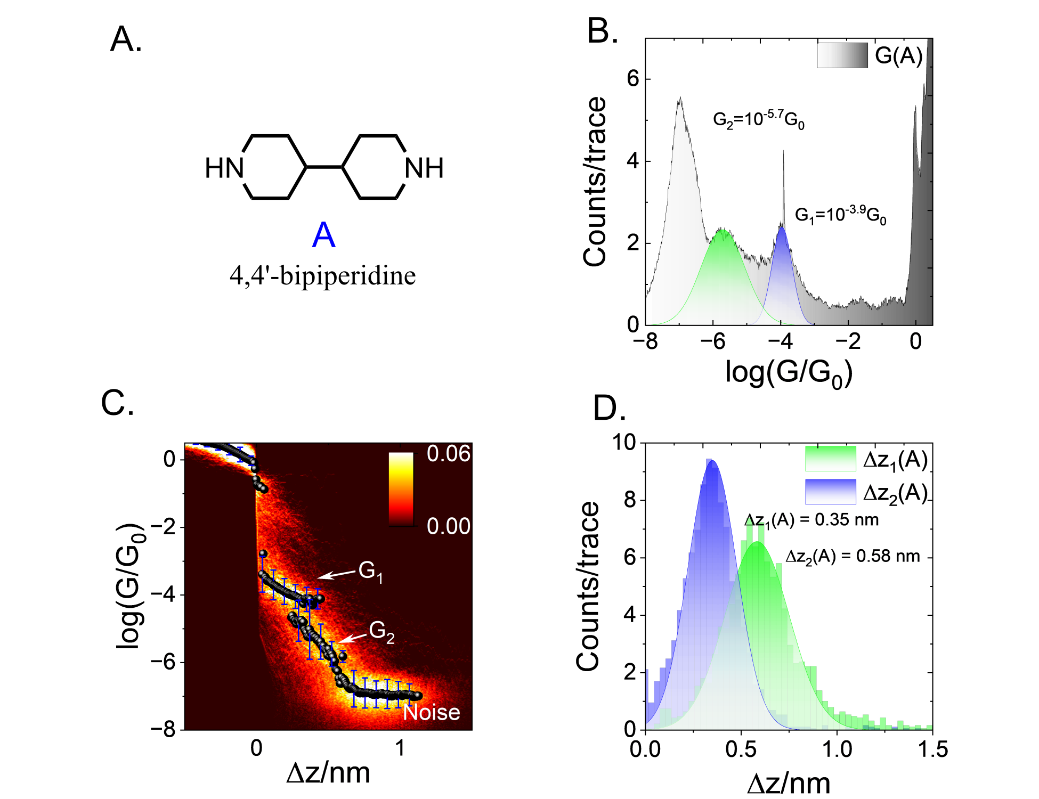


**Figure S2.** Low concentration measurement of molecule A **(**Measured using 2 μM molecular solution)**. A.** Molecular structure of 4,4'-bipiperidine-A molecule. **B.** 1D conductance **C.**2D conductance-distance and **D.** plateau length histograms. (In 2D conductance histogram, the black balls represent the Gaussian peak maximum at each of the x values, and black error bars represent the standard deviation derived from the Gaussian fitting.

**
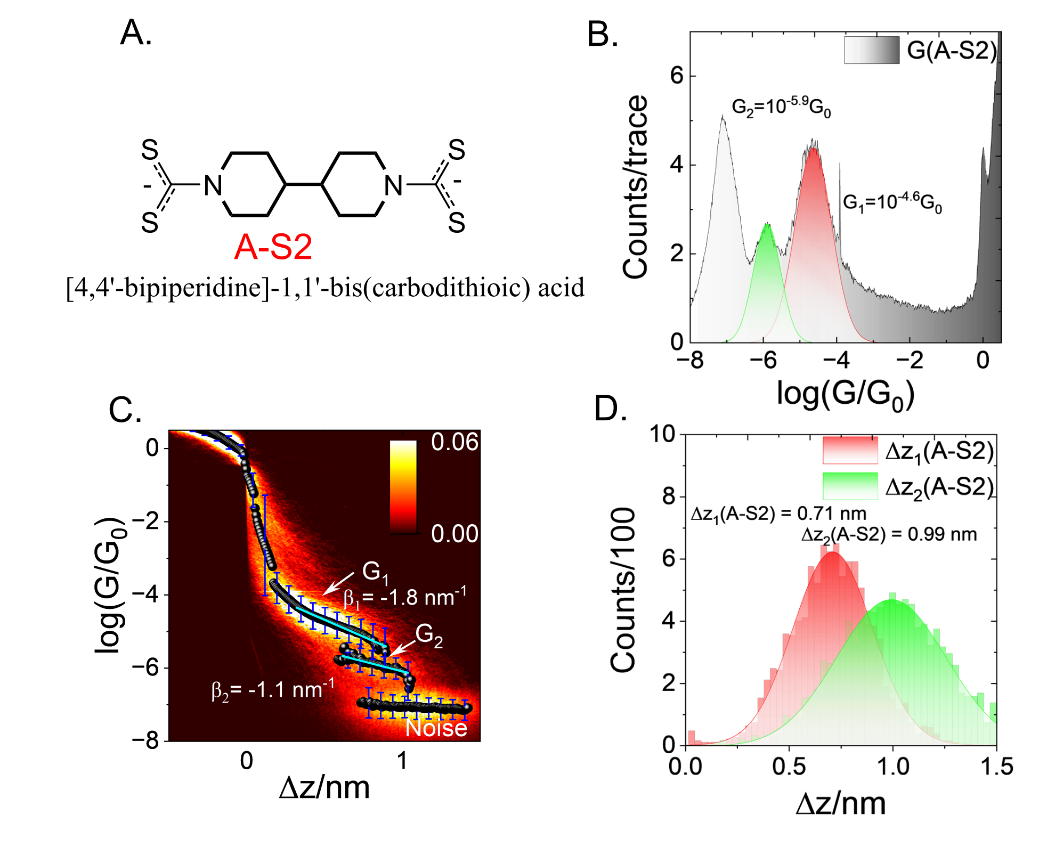
**

**Figure S3. A.** Molecular structure of A-S2 molecule. **B.** 1D conductance **C.**2D conductance-distance and **D.** plateau length histograms**.** (In 2D conductance histogram, the black balls represent the Gaussian peak maximum at each of the x values, and black error bars represent the standard deviation derived from the Gaussian fitting. Purple line represents the linear fit giving the decay factor(β) represented in the plots.


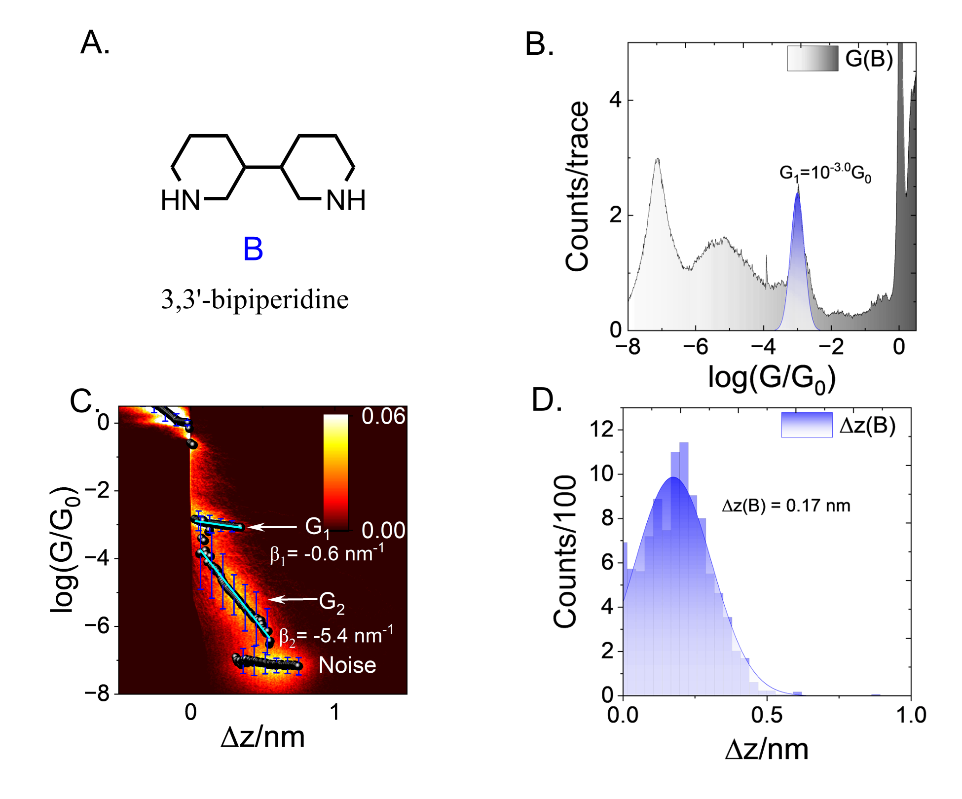


**Figure S4. A.** Molecular structure of 3,3'-bipiperidine-B molecule. **B.** 1D conductance **C.**2D conductance-distance and **D.** plateau length histograms**.** (In 2D conductance histogram, the black balls represent the Gaussian peak maximum at each of the x values, and black error bars represent the standard deviation derived from the Gaussian fitting. Purple line represents the linear fit giving the decay factor(β) represented in the plots.


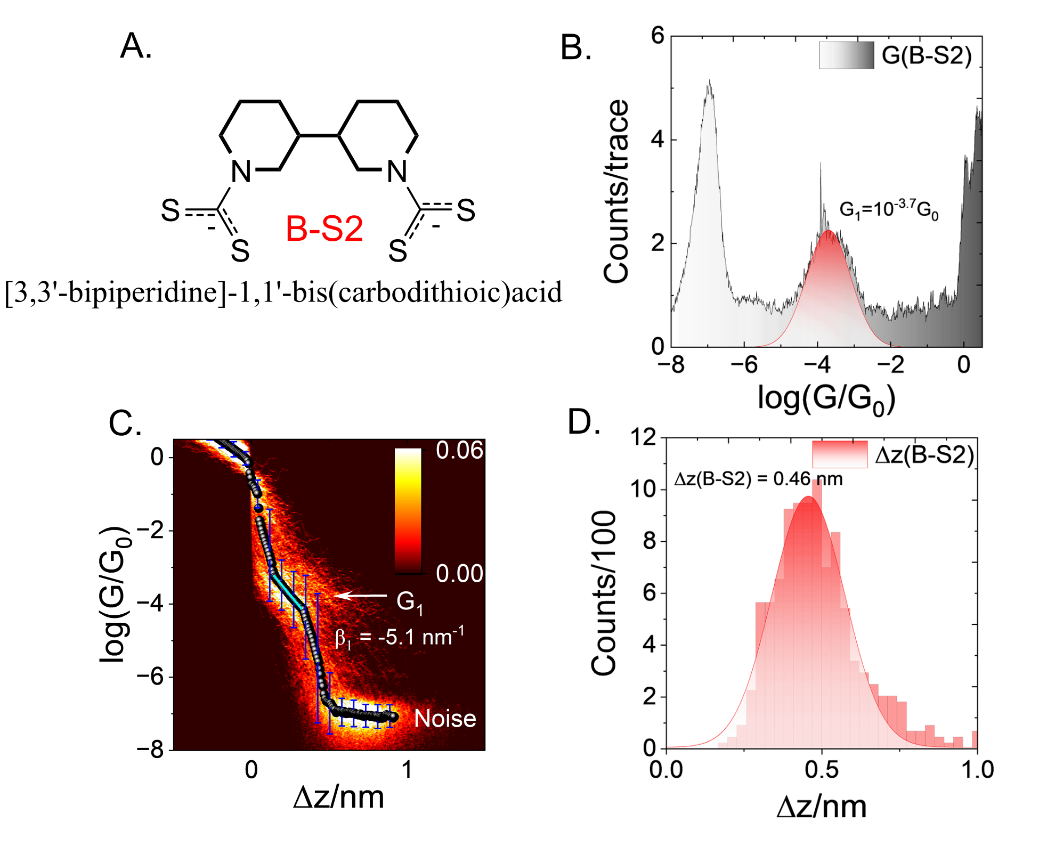


**Figure S5. A.** Molecular structure of B-S2 molecule. **B.** 1D conductance **C.**2D conductance-distance and **D.** plateau length histograms**.** (In 2D conductance histogram, the black balls represent the Gaussian peak maximum at each of the x values, and black error bars represent the standard deviation derived from the Gaussian fitting. Purple line represents the linear fit giving the decay factor(β) represented in the plots.


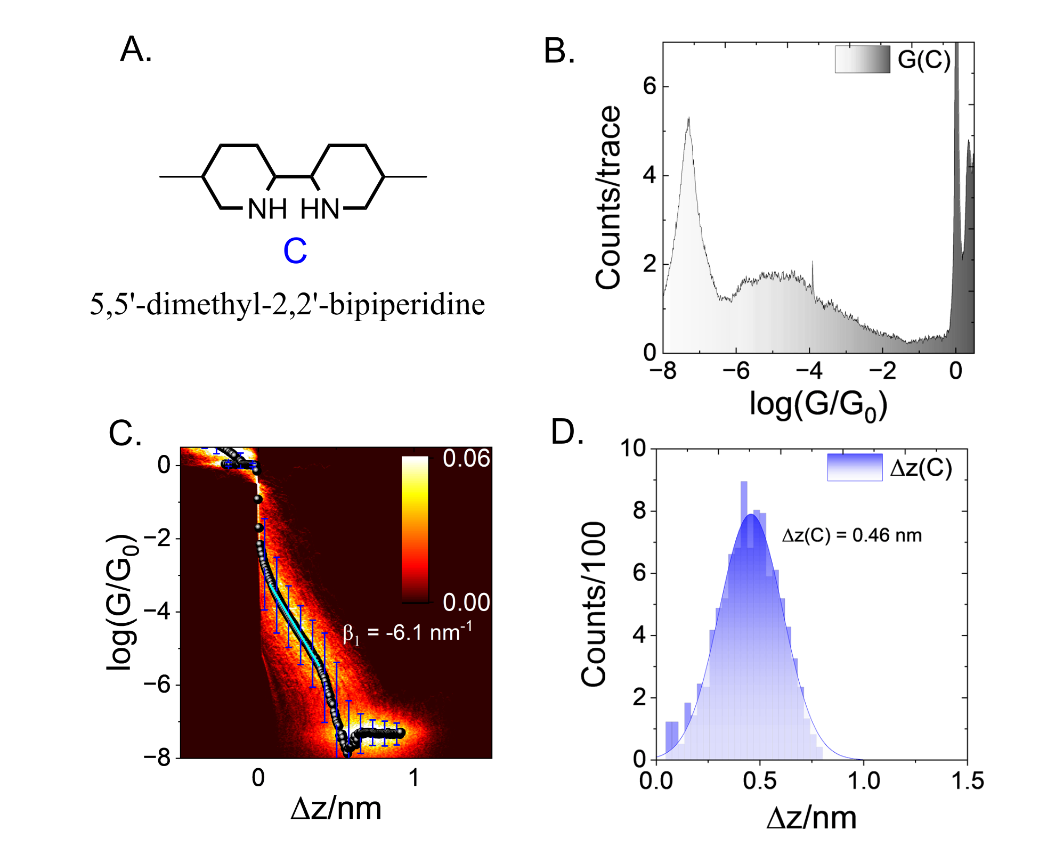


**Figure S6. A.** Molecular structure of 5,5'-dimethyl-2,2'-bipiperidine-C molecule. **B.** 1D conductance **C.**2D conductance-distance and **D.** plateau length histograms**.** (In 2D conductance histogram, the black balls represent the Gaussian peak maximum at each of the x values, and black error bars represent the standard deviation derived from the Gaussian fitting. Purple line represents the linear fit giving the decay factor(β) represented in the plots.


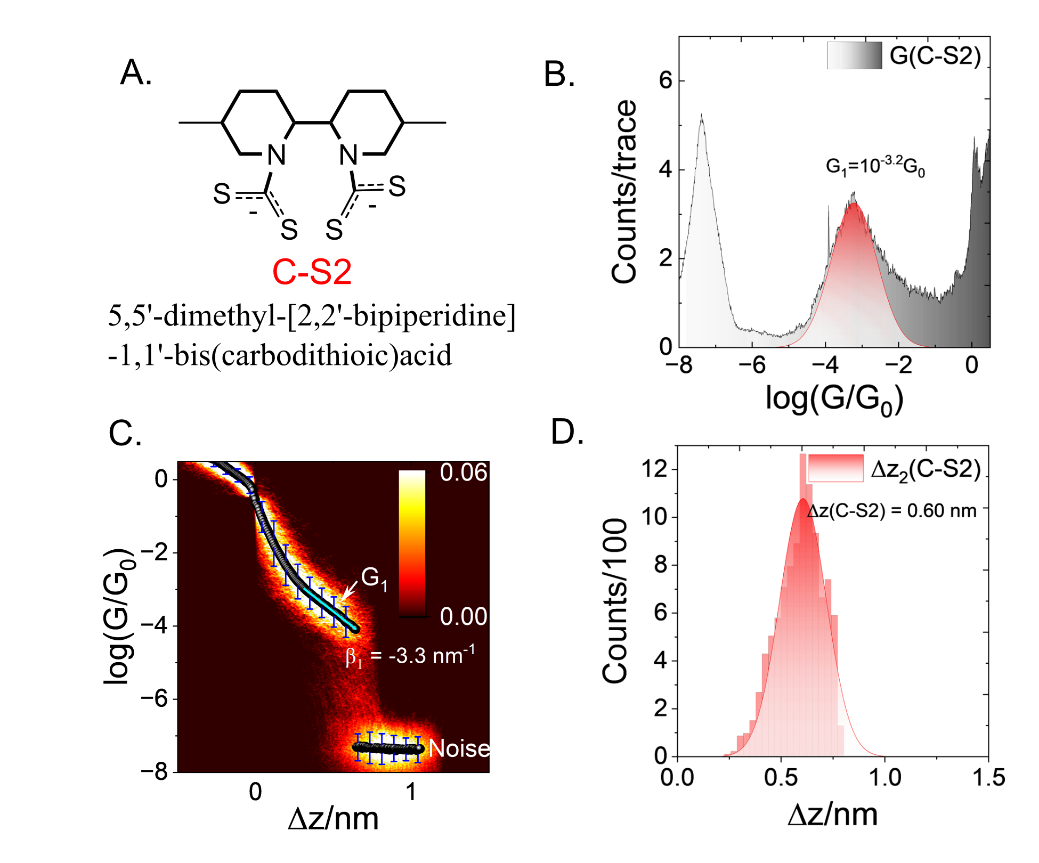


**Figure S7. A.** Molecular structure of C-S2 molecule. **B.** 1D conductance **C.**2D conductance-distance and **D.** plateau length histograms**.** (In 2D conductance histogram, the black balls represent the Gaussian peak maximum at each of the x values, and black error bars represent the standard deviation derived from the Gaussian fitting. Purple line represents the linear fit giving the decay factor(β) represented in the plots.


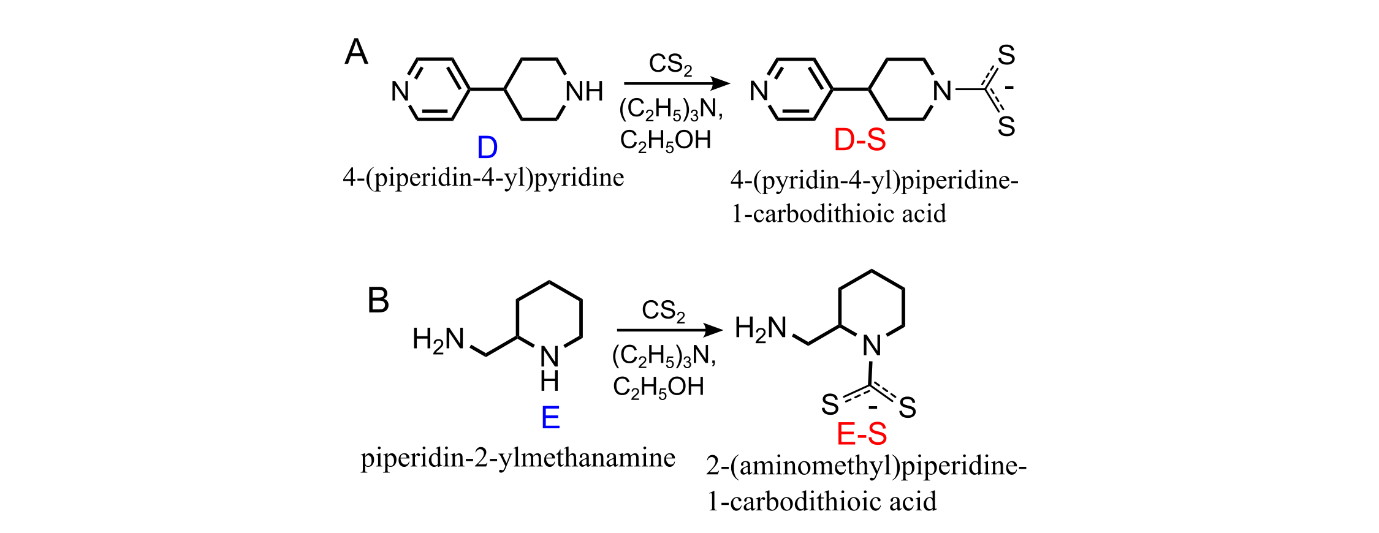


**Figure S8.** Dithiocarbamylation of **A.** 4-(piperidin-4-yl)pyridine-D. **B.** piperidin-2-yl-methylamine-E.


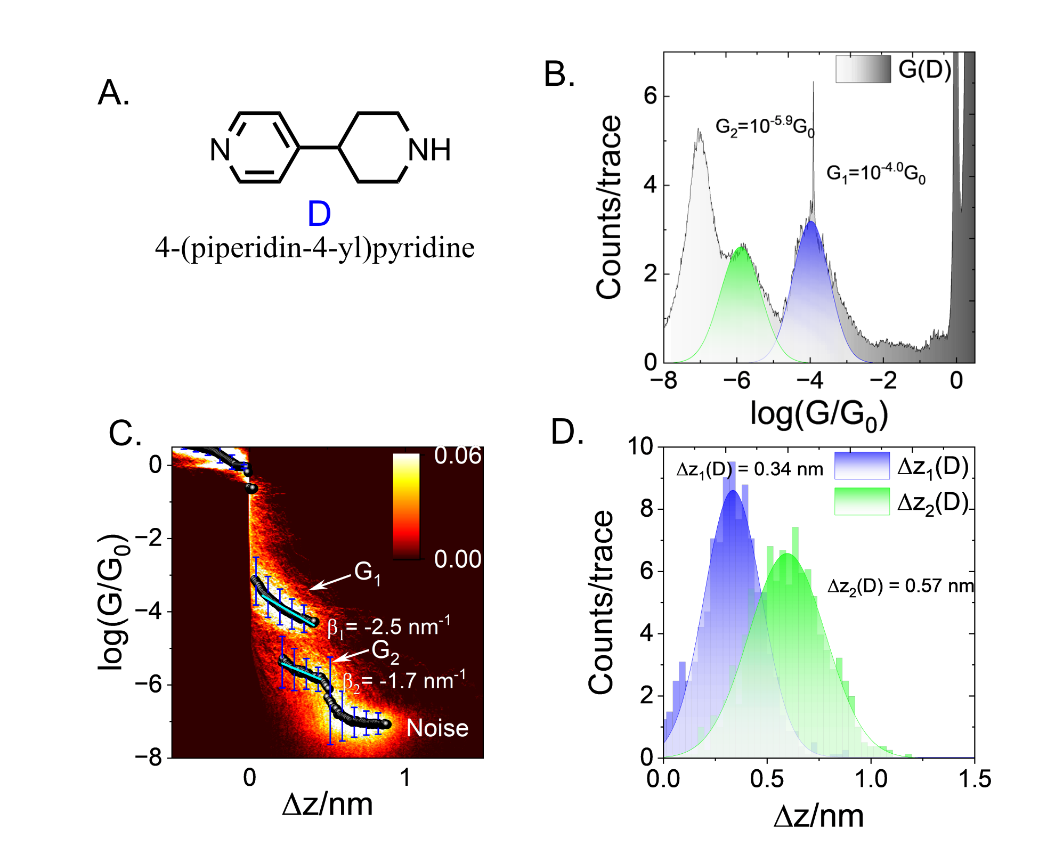


**Figure S9. A.** Molecular structure of 4-(piperidin-4-yl)pyridine-D molecule. **B.** 1D conductance **C.**2D conductance-distance and **D.** plateau length histograms**.** (In 2D conductance histogram, the black balls represent the Gaussian peak maximum at each of the x values, and black error bars represent the standard deviation derived from the Gaussian fitting. Purple line represents the linear fit giving the decay factor(β) represented in the plots.


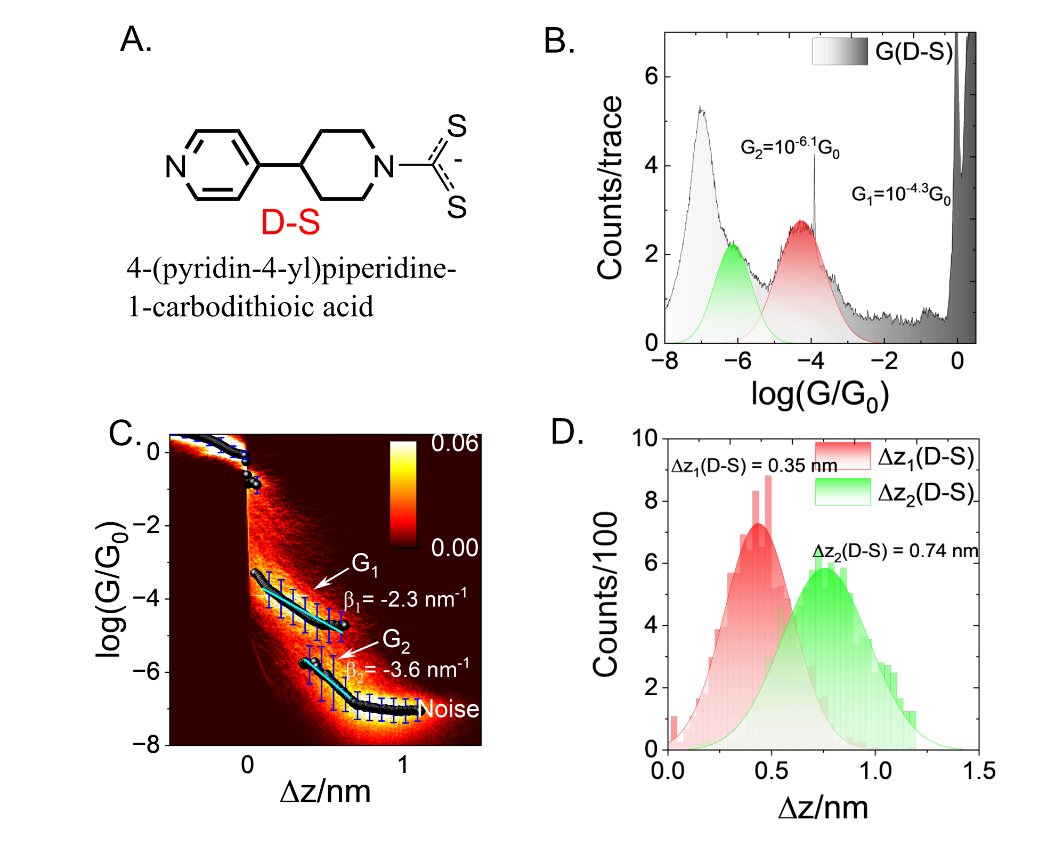


**Figure S10. A.** Molecular structure of D-S molecule. **B.** 1D conductance **C.**2D conductance-distance and **D.** plateau length histograms**.** (In 2D conductance histogram, the black balls represent the Gaussian peak maximum at each of the x values, and black error bars represent the standard deviation derived from the Gaussian fitting. Purple line represents the linear fit giving the decay factor(β) represented in the plots.


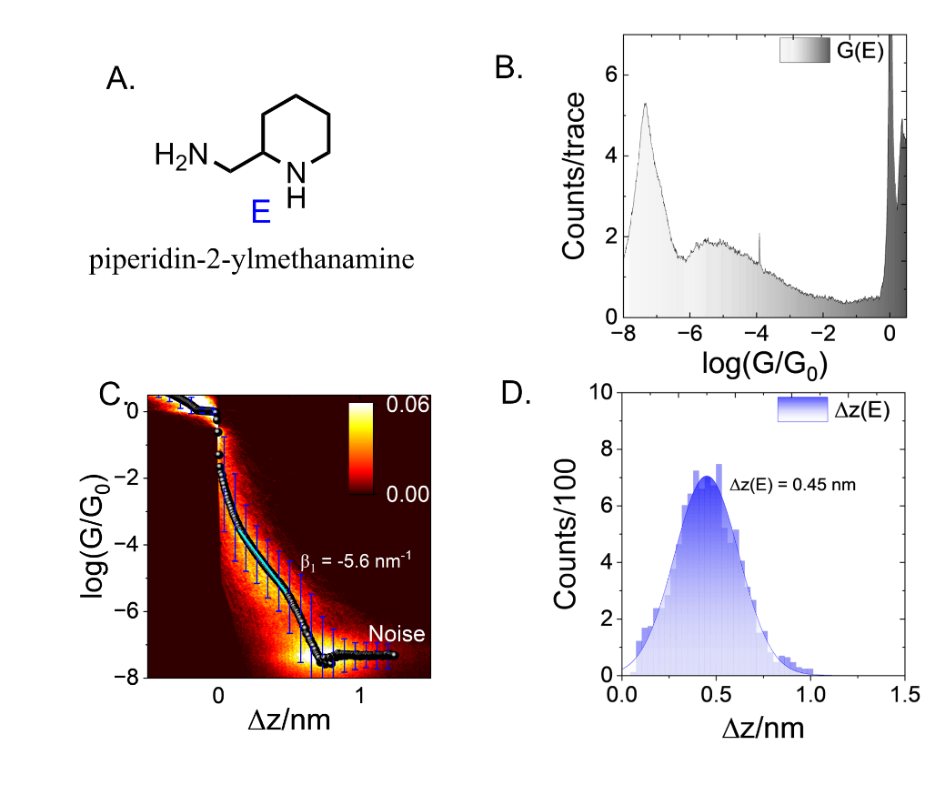


**Figure S11. A.** Molecular structure of piperidin-2-yl-methylamine-E molecule. **B.** 1D conductance **C.**2D conductance-distance and **D.** plateau length histograms**.** (In 2D conductance histogram, the black balls represent the Gaussian peak maximum at each of the x values, and black error bars represent the standard deviation derived from the Gaussian fitting. Purple line represents the linear fit giving the decay factor(β) represented in the plots.

**
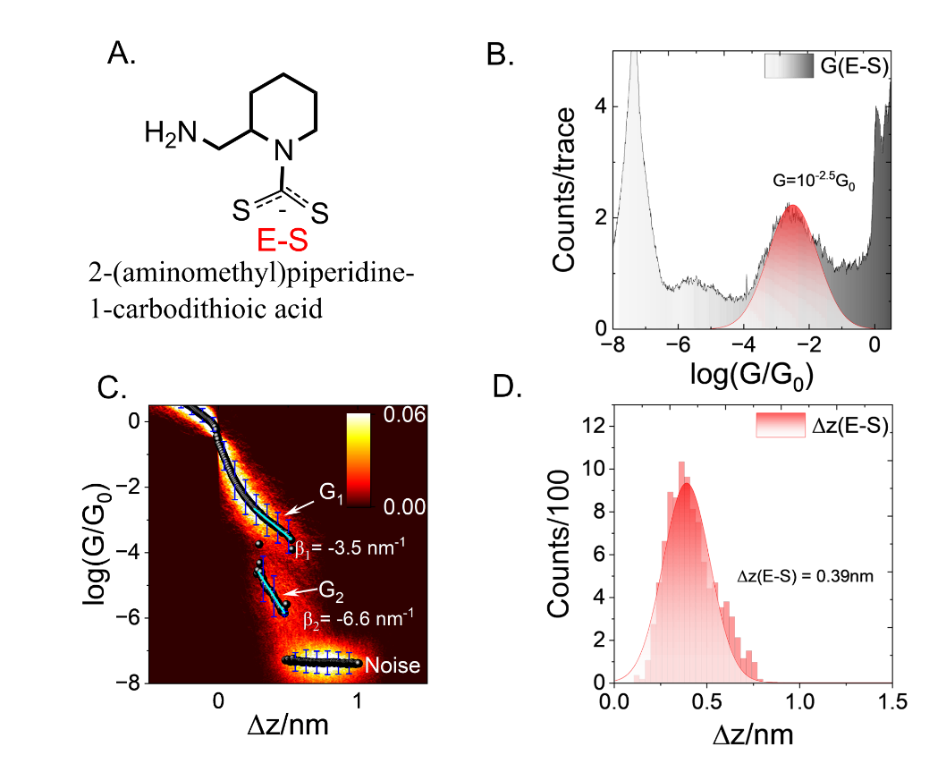
**

**Figure S12. A.** Molecular structure of E-S molecule. **B.** 1D conductance **C.**2D conductance-distance and **D.** plateau length histograms**.** (In 2D conductance histogram, the black balls represent the Gaussian peak maximum at each of the x values, and black error bars represent the standard deviation derived from the Gaussian fitting. Purple line represents the linear fit giving the decay factor(β) represented in the plots.

1. **Computational studies**
   1. **Computational methods**

The optimized geometry and ground state Hamiltonian and overlap matrix elements of each structure (as shown in Figure 4) was self-consistently obtained using the SIESTA implementation^[5]^ of density functional theory (DFT). SIESTA employs norm-conserving pseudo-potentials to account for the core electrons and linear combinations of atomic orbitals to construct the valence states. The generalized gradient approximation (GGA) of the exchange and correlation functional is used with the Perdew-Burke-Ernzerhof parameterization (ca) a double-ζ polarized (DZP) basis set, a real-space grid defined with an equivalent energy cut-off of 250 Ry. The geometry optimization for each structure is performed to the forces smaller than 10 meV/Å.

The mean-field Hamiltonian obtained from the converged DFT calculation was combined with Gollum^[6,7]^ implementation of the non-equilibrium Green’s function method^[7]^ to calculate the phase-coherent, elastic scattering properties of the each system consist of left gold (source) and right gold (drain) leads and the scattering region. Each electrode consists 136 gold atoms with 111 surface configurations (facing the molecule) and periodic in transverse directions. The transmission coefficient *T(E)* for electrons of energy *E* (passing from the source to the drain) is calculated via the relation: $T\left( E \right)=Trace\left( \Gamma_{R}(E)G^{R}(E)\Gamma_{L}(E)G^{R\dagger}(E) \right)$. In this expression,$\Gamma_{L,R}\left( E \right)=i\left( \sum_{L,R}\left( E \right)-{\sum_{L,R}}^{\dagger}\left( E \right) \right)$ describe the level broadening due to the coupling between left (L) and right (R) electrodes and the central scattering region, $\sum_{L,R}\left( E \right)$are the retarded self-energies associated with this coupling and $G^{R}=\left( ES-H-\sum_{L}-\sum_{R} \right)^{-1}$ is the retarded Green’s function. The electrical conductance is then calculated using the Landauer formula $G=G_{0}\int dE T(E) (-\partial f(E,T)/\partial E)$where *G_0_* = 2$e^{2}$/*h* is the conductance quantum, *f(E)* *= (1 + exp((E −* $E_{F}$*)/*$K_{B}$*T)*$)^{-1}$ the Fermi-Dirac probability distribution function, *T* is the temperature, *E_F_* is the Fermi energy, is the conductance quantum, *e* is electron charge and *h* is the Planck’s constant. Our DFT calculations in the main text show qualitative agreement with our measurements across the entire energy range between the HOMO and LUMO. Comparing the calculated conductance with the measured values suggests that the transport is in the cotunneling regime. Therefore, we have chosen a wide Fermi energy range that does not include HOMO and LUMO resonances for all sets of molecules shown in Figure 4D. The tight-binding (TB) model is constructed using a Hückel Hamiltonian derived from DFT calculations, as illustrated in Figure 5A of the main text. To obtain TB parameters, we have performed DFT calculations with minimal basis size (SZ) of the isolated molecules and obtained the on-site energies and coupling integrals between different orbitals and used similar ratios in the TB model. In this TB model, single σ orbitals interact with their first (γ₁), second (γ₂), and third (γ₃) nearest neighbors, where γ₁ = 0.8γ, γ₂ = 0.6γ, γ₃ = 0.4γ, and γ = -2 eV represents the coupling strength between orbitals in the leads. The on-site energies in the leads are set to zero, and the on-site energy for the σ orbitals is adjusted to align the Fermi level with the center of the TB HOMO-LUMO gap. To assess the robustness of the QI pattern observed with this TB model, we introduced 15% variations in γ₁, γ₂, and γ₃ and generated a histogram using the method discussed in ^[8]^.

- 1. **Supplementary computational figures, tables and discussion**

**Figure S13. Effect of rotation angle between molecular and electrode.** (a) Molecular structures of Dithiocarbamylation of 4,4`-bipiperidine-A between two gold electrodes. (b) Transmission coefficients for various configurations with different dihedral angles (|∆𝜃| = $6^{0}$). (c) Histogram of transmission coefficients over a range of molecule-electrode dihedral angles for the selected 𝐸_𝐹_​ (gray area).

**Figure S14. Effect of rotation angle between molecular and electrode**. (a) Molecular structures of Dithiocarbamylation [4,4`-bipiperidine]-1,1`-bis(carbodithioic) acid A-S2 between two gold electrodes. (b) Transmission coefficient for various configurations with different dihedral angles (|∆𝜃| = $3^{0}$). (c) Histogram of transmission coefficients over a range of molecule-electrode dihedral angles for the selected 𝐸_𝐹_​ (gray area).

**Figure S15. Effect of Molecular Structure on Energy Variations.** (a) Chemical structures of five molecular systems (A–E). (b) Optimized geometries of the molecules between gold electrodes. (c) Energy variations (Δ𝐸) as a function of dihedral angle (Δθ) with the rotating part highlighted in gray (for each molecular junction, highlighting the structural influence on electronic properties. Insets show the molecular structures within the junctions.

**Figure S16. Influence of Dihedral Angle on Electron Transport.** (a) Chemical structures of five molecular systems (A–E), with the rotating part highlighted in gray. (b) Optimized geometries of the molecules between gold electrodes. (c) Transmission coefficient as a function of energy (𝐸) for two different dihedral angles (𝜃_1_ and 𝜃_2_), showing the effect of molecular conformation on electronic transport properties. Insets illustrate the corresponding molecular structures for each dihedral angle.

**Figure S17. Effect of Molecular Structure on Energy Variations.** (a) Chemical structures of five molecular structures obtained after dithiocarbamylation (A-S2 to E-S). (b) Optimized geometries of the molecules between gold electrodes. (c) Energy variations (Δ𝐸) as a function of dihedral angle (Δθ) with the rotating part highlighted in gray (for each molecular junction, highlighting the structural influence on electronic properties. Insets show the molecular structures within the junctions.

**Figure S18. Influence of Dihedral Angle on Electron Transport.** (a) Chemical structures of five molecular structures obtained after dithiocarbamylation (A-S2 to E-S), with the rotating part highlighted in gray. (b) Optimized geometries of the molecules between gold electrodes. (c) Transmission coefficient as a function of energy (𝐸) for two different dihedral angles (𝜃_1_ and 𝜃_2_), showing the effect of molecular conformation on electronic transport properties. Insets illustrate the corresponding molecular structures for each dihedral angle.


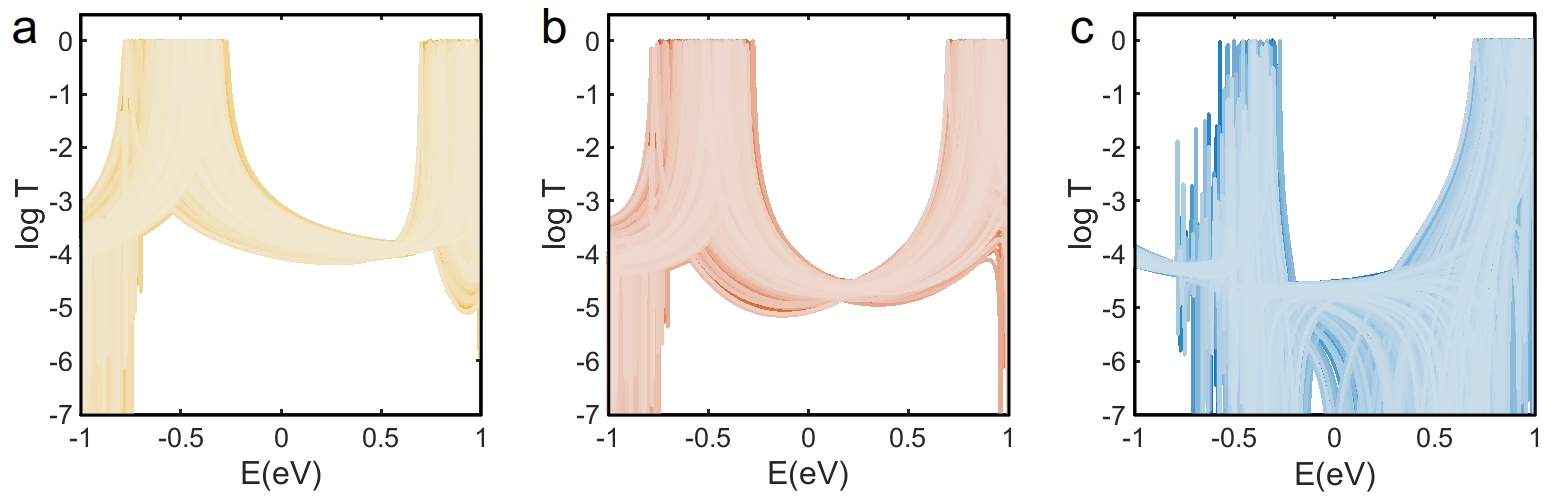


**Figure S19. TB transmission function for bicyclohexyl with 15% tolerance in TB parameters.** (a) ortho, (b) meta, and (c) para connectivities.

**Figure S20. DFT analysis of conformational effects.** (a) Relative total energies (ΔE) for the chair and boat conformations in planar and tilted geometries. ΔE is referenced to the corresponding chair geometry; the chair is the ground state, lower by c.a. 600 meV compared with the boat. (b) Transmission coefficients for the four configurations: chair–planar, chair–tilted, boat–planar, and boat–tilted between electrodes.

**Table S1.** Frontier orbitals of relaxed structure of different molecules with amine as anchoring groups from HOMO-2 to LUMO+2 orbitals, the energy unit is eV.

**Table S2.** Frontier orbitals of relaxed structure of different molecules with dithiocarbamate as anchoring group from HOMO-2 to LUMO+2 orbitals, the energy unit is eV.

**Table S3.** Frontier orbitals of relaxed structure of π-conjugated systems molecules from HOMO-2 to LUMO+2 orbitals, the energy unit is eV.

1. **References:**

[1] U. Rashid, E. Chatir, L. Medrano Sandonas, P. Sreelakshmi, A. Dianat, R. Gutierrez, G. Cuniberti, S. Cobo, V. Kaliginedi, “Dithienylethene‐Based Single Molecular Photothermal Linear Actuator” *Angew. Chem. Int. Ed.* **2023**, *62*, e202218767.

[2] U. Rashid, W. Bro-Jørgensen, K. Harilal, P. Sreelakshmi, R. R. Mondal, V. Chittari Pisharam, K. N. Parida, K. Geetharani, J. M. Hamill, V. Kaliginedi, “Chemistry of the Au–Thiol Interface through the Lens of Single-Molecule Flicker Noise Measurements” *J. Am. Chem. Soc.* **2024**, *146*, 9063–9073.

[3] U. Rashid, L. Medrano Sandonas, E. Chatir, Z. Ziani, P. Sreelakshmi, S. Cobo, R. Gutierrez, G. Cuniberti, V. Kaliginedi, “Mapping the Extended Ground State Reactivity Landscape of a Photoswitchable Molecule at a Single Molecular Level” *J. Am. Chem. Soc.* **2025**, *147*, 830–840.

[4] U. Rashid, Single Molecular Break Junctions: Insights into Charge Transport and Chemical Reactivity, PhD thesis, Indian Institute of Science, **2025**.

[5] J. M. Soler, E. Artacho, J. D. Gale, A. García, J. Junquera, P. Ordejón, D. Sánchez-Portal, “The SIESTA method for *ab initio* order- *N* materials simulation” *J. Phys. Condens. Matter* **2002**, *14*, 2745–2779.

[6] H. Sadeghi, “Theory of electron, phonon and spin transport in nanoscale quantum devices” *Nanotechnology* **2018**, *29*, 373001.

[7] J. Ferrer, C. J. Lambert, V. M. García-Suárez, D. Z. Manrique, D. Visontai, L. Oroszlany, R. Rodríguez-Ferradás, I. Grace, S. W. D. Bailey, K. Gillemot, H. Sadeghi, L. A. Algharagholy, “GOLLUM: a next-generation simulation tool for electron, thermal and spin transport” *New J. Phys.* **2014**, *16*, 093029.

[8] A. Daaoub, L. Ornago, D. Vogel, P. Bastante, S. Sangtarash, M. Parmeggiani, J. Kamer, N. Agraït, M. Mayor, H. Van Der Zant, H. Sadeghi, “Engineering Transport Orbitals in Single-Molecule Junctions” *J. Phys. Chem. Lett.* **2022**, *13*, 9156–9164.
